# Advances in the Utilization of Tea Polysaccharides: Preparation, Physicochemical Properties, and Health Benefits

**DOI:** 10.3390/polym14142775

**Published:** 2022-07-06

**Authors:** Qian Wang, Xiaoyan Yang, Changwei Zhu, Guodong Liu, Yujun Sun, Lisheng Qian

**Affiliations:** 1 College of Life and Health Sciences, Anhui Science and Technology University, Chuzhou 233100, China; qianwang420@163.com (Q.W.); zhucw@ahstu.edu.cn (C.Z.); liugd@ahstu.edu.cn (G.L.); 2 College of Agriculture, Anhui Science and Technology University, Chuzhou 233100, China; yangxiaoyan8166@163.com

**Keywords:** tea, polysaccharides, extraction method, chemical composition, bioactivity, gut microbiota

## Abstract

Tea polysaccharide (TPS) is the second most abundant ingredient in tea following tea polyphenols. As a complex polysaccharide, TPS has a complex chemical structure and a variety of bioactivities, such as anti-oxidation, hypoglycemia, hypolipidemic, immune regulation, and anti-tumor. Additionally, it shows excellent development and application prospects in food, cosmetics, and medical and health care products. However, numerous studies have shown that the bioactivity of TPS is closely related to its sources, processing methods, and extraction methods. Therefore, the authors of this paper reviewed the relevant recent research and conducted a comprehensive and systematic review of the extraction methods, physicochemical properties, and bioactivities of TPS to strengthen the understanding and exploration of the bioactivities of TPS. This review provides a reference for preparing and developing functional TPS products.

## 1. Introduction

As a traditional drink, tea has been cultivated and consumed for thousands of years, and it is deeply loved by consumers from many countries, such as China, Japan, and South Korea. Tea not only creates a lot of wealth but also generates tea culture and tea ceremony [1]. As a result, tea has become one of the most popular beverages in the world after water [2,3,4].

The unprecedented popularity of tea is due not only to its unique aroma and taste but also to the health benefits of drinking it. The primary bioactivities of tea, including anti-oxidation, hypoglycemic, antibacterial, hypolipidemic, and anti-cancer activities, have been studied and explored. Tea has also been broadly utilized in the food, medical, and health care industries [5,6]. Tea’s biological and pharmacological activities are mainly attributed to the diversity of its chemical components. The chemical features of tea mainly include tea polyphenols (TPPs), tea polysaccharides (TPSs), tea proteins, catechins, theanine, and inorganic elements [4]. Tea polyphenols have long received attention for their excellent antioxidant properties for which accumulating evidence has been presented [7]. Modern pharmacological studies have shown that TPS, an important bioactive component along with TPP, is also the main tea compound that helps lower blood glucose and lipids, resist oxidation, and enhance the body’s immune function [8,9,10]. It also has excellent potential for development and application in the cosmetic industry [11]. In general, the content of TPS decreases with increases in tea quality or grade [12]. Wang et al., reported that the TPS content in low-grade tea was twice that of high-grade tea [13]. Therefore, using low-grade tea as a raw material to extract TPS is conducive to the full utilization of tea resources and has important implication for preventing diseases and promoting human health.

Therefore, the authors of this paper conducted a detailed comparison and summary of the current research on tea polysaccharide’s extraction, preliminary physicochemical properties, and in vitro and in vivo bioactivities in order to provide new insights for the better utilization and development of TPS or TPS-related functional foods.

## 2. TPS Extraction

**Figure 1:**
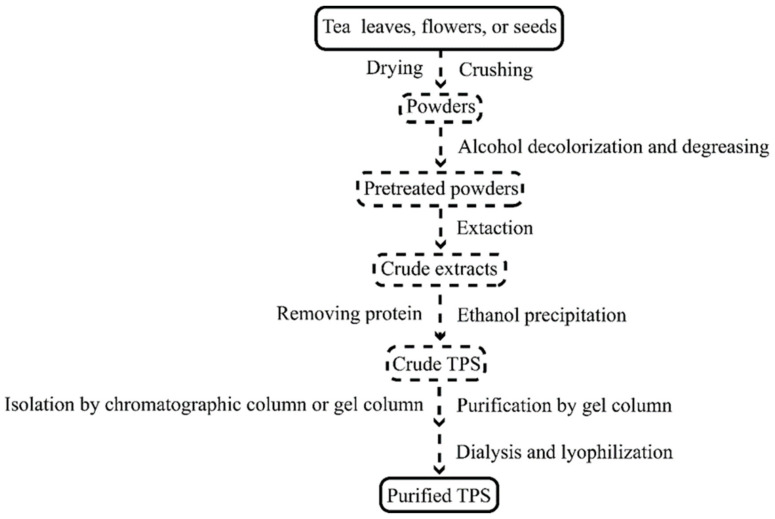
The conventional process of TPS preparation.

Tea leaves, flowers, and seeds are the three primary sources of TPS extraction materials. The current production process of TPS mainly includes hot water extraction, ultrasonic-assisted extraction, microwave-assisted extraction, and enzymolysis extraction (Table 1). Its conventional preparation process is shown in Figure 1.

### 2.1. Hot Water Extraction

Most bioactive polysaccharides are polar, so polar solvents such as hot water or alkaline solutions are usually used for polysaccharide extraction [33]. Hot water extraction is a classic method widely used to prepare polysaccharides in food, medicine, and other industries [34]. Chen et al., used water bath heating (70 °C, 60 min) to extract three kinds of crude TPSs from black, oolong, and green tea leaves [35]. Xu et al., prepared TPS from Pu-erh tea three times for 180 min in hot water at 70 °C [36]. Fan et al., extracted TPS twice in Fuan Baicha and Pingyang Tezaocha by adding double-distilled water and heating in a water bath at 80 °C for 1.5 h [37]. Zhu et al., used the response surface methodology to explore the extraction process of Fuzhuan tea crude polysaccharide (CDTPS) and found that the optimal extraction conditions (repeated four times) were as follows: an extraction time of 2 h, a solid–liquid ratio of 1:20, and an extraction temperature of 95 °C. Under these conditions, the yield of CDTPS was 6.07% [10]. The response surface methodology used by Jin et al., predicted the optimal extraction conditions of TPS via repetition four times in white tea: the optimal extraction time was 97.8 min, the extraction temperature was 54.1 °C, and the material–water ratio was 12.48 L/g [14]. Wang et al., pretreated dried green tea leaves and flowers in 95% ethanol and 40 °C for 2 h, then repeated the process three times to remove pigments and other substances. Then, 2 L of distilled water was added to the filtered tea samples for extraction in a water bath at 60 °C for 2 h. After filtration, 2.5 L of distilled water was added, and the hot water extraction was repeated again (60 °C, 2 h) [38]. Similarly, Cai et al., pretreated green tea leaves with absolute ethanol for 24 h to remove some small-molecular pigments and polyphenols, and then they dried the tea samples with deionized water for 90 min at 60 °C [16]. Li et al., also pretreated Chin brick tea powder with 80% ethanol, centrifuged it, and then continuously stirred it with distilled water (1:20, *w*/*v*) for 2 h at 90 °C to extract TPS [17]. Qin et al., pretreated Liupao tea samples with 80% ethanol for 24 h. After filtration and drying, the samples were extracted with deionized water at 70 °C for 2 h, and the process was repeated three times [18]. Wei et al., performed the hot water extraction of dried tea flower polysaccharides (TFPSs) and then extracted TFPSs twice with distilled water (1 h each). They found that the yield of TFPS increased with the extraction temperature, and 90 °C was the optimal extraction temperature for TFPS. The yield at this condition was close to 35% [19]. Though hot water extraction is a commonly used method for TPS extraction, conventional hot water extraction has disadvantages such as a low extraction efficiency, long extraction time, and high extraction temperature, all of which limit its availability [33,39]. For example, Wang et al., further compared the yields of hot water extraction, boiling water extraction, and enzymolysis extraction for TFPS, and they found that the yield of TFPS obtained with enzymolysis extraction was the highest (2.01%), followed by boiling water extraction (1.91%) and finally hot water extraction (1.83%) [20]. Zhu et al., compared the yields of crude green tea polysaccharides (CTPSs) under hot water extraction (WE), enzymatic extraction (EE), microwave-assisted extraction (MAE) and ultrasonic-assisted extraction (UAE), and they found that the four yields of CTPS under these extraction methods were 3.98%, 4.17%, 4.31%, and 4.52%, respectively [21]. Numerous studies have verified that although hot water extraction has strong practicability, its obtained TPS yield is relatively low and easily leads to the unnecessary waste of raw materials. Therefore, many researchers have also improved the technology on the basis of hot water extraction and developed other auxiliary extraction methods, such as ultrasonic-assisted extraction, microwave-assisted extraction, and enzyme-assisted extraction, to improve the extraction efficiency of TPS [40].

### 2.2. Ultrasonic-Assisted Extraction (UAE)

UAE can accelerate the rupture of plant cell walls by the high-speed movement of molecules in samples caused by high-frequency ultrasonic vibration, thereby dissolving and releasing intracellular substances. Karadag et al., used UAE to extract low-grade green tea polysaccharides (GTPSs) and then reported the optimal extraction parameters through response surface optimization as follows: 80 °C for extraction temperature, 60 min for extraction time, 400 W for ultrasonic power, and 22 mL/g for liquid–solid ratio. Under these conditions, the yield of GTPS was 4.65%, which was higher than that of the hot water extraction method (1.83%) without ultrasound [25]. In addition, they also found that the Mw of GTPS obtained with ultrasonic-assisted extraction was lower, which may have been due to the partial degradation of TPS caused by the ultrasonic process. Zhu et al., prepared TPS from coarse green tea leaves, placing the tea leaves in an ultrasonic bath (50 °C, 200 W) for pretreatment for 30 min and then performing extraction in a water bath for 90 min. The TPS yield obtained with this method was higher than other tested methods [21]. To explore the effects of ultrasound on the structure and activity of yellow tea polysaccharide (YTPS), Wang et al., treated a YTPS fraction obtained after hot water extraction and deproteinization with ultrasound (20 kHz, 500 W) for 55 min. The results showed that ultrasonic treatment basically did not change the main chemical composition of YTPS but did cause it to degrade [26]. Wei et al., mixed dried green tea flower blocks with distilled water and extracted them for 5 min at 25 °C under ultrasonic powers of 100, 150, 200, 250, and 300 W. This process was repeated twice to obtain crude TFPS [19]. Overall, the UAE method has the advantages of saved time, simple operation, experimental safety, low cost, and high extraction rate. Still, it may degrade soluble TPS and affect its bioactivity.

### 2.3. Microwave-Assisted Extraction (MAE)

Recently, microwave-assisted extraction (MAE) technology has become widely used to analyze and extract active components in plants. MAE is a new extraction technology that uses high-frequency electromagnetic waves (0.3–300 GHZ) with strong penetrability and heating effect to extract active plant components. High-energy microwaves can penetrate solvents and plant cell walls, transfer energy to the cytoplasm, and interact with polar components to generate heat, which increases the temperature and pressure inside cells. When the pressure reaches a certain level, the cell wall expands and ruptures, releasing intracellular polysaccharides and other substances [41]. Shuntaro et al., used MAE technology to extract TPS from tea residues (green tea, black tea, and oolong tea). When the extraction conditions were a solid/liquid ratio of 1:20, an extraction temperature of 200–230 °C, and an extraction time of 2 min, the yield of tea residue TPS was 40–50% [27]. Wei et al., used MAE equipment to extract TFPS twice, for 5 min each time. They found that the yield of TFPS changed irregularly with the increase in microwave power. In addition, with increases in microwave power, the content of neutral sugars in TFPS increased while the content of acidic sugars increased and then decreased [19]. Li et al., used a 600 W microwave instrument to extract coarse green tea crude TPS (CTPS), and the extraction process was repeated three times. After extraction with MAE, the content of soluble protein in CTPS was the highest of all tested methods, reaching 5.93%. Furthermore, they found that MAE treatment had little effect on CTPS chains with high Mw but resulted in the drastic degradation of small-Mw CTPS. According to related reports, small-Mw polysaccharides tend to have better bioactivities than their high-Mw counterparts [42]. The subsequent in vitro activity test of CTPS prepared by the MAE method by Zhu et al., also confirmed this conclusion [21]. Compared to other extraction methods, the MAE method has the advantages of high extraction efficiency, high purity, non-degradable active ingredients, convenient operation, saved time, and environmental friendliness. It is a “green extraction process”, which has made it popular. Although MAE has favorable prospects in TPS extraction, it also has disadvantages such as complex extract components, difficult separation and purification in the later stages, and the necessity of polar solvents [43]. Therefore, in addition to the basic closed and open systems, several improved microwave extraction technologies, such as vacuum microwave-assisted extraction, nitrogen-protected microwave-assisted extraction, ultrasonic microwave-assisted extraction, and dynamic microwave-assisted extraction, have been developed [41].

### 2.4. Enzymolysis Extraction

The enzymolysis method refers to the destruction of plant cell walls with enzymatic hydrolysis. The cell wall is decomposed into small molecular substances readily soluble in the extraction solvent, thereby accelerating the dissolution of active ingredients. The yield of TPS extracted with enzymatic hydrolysis is usually higher and the effect of mixed enzymes is better than that of a single enzyme. However, the enzyme’s activity is easily affected by the reaction temperature, pH, and concentration, so the requirements for experimental conditions and costs are usually higher. Baik et al., investigated the effect of the simultaneous treatment of pectinase and tannase on TPS extraction from green tea. They found that the concurrent treatment of the two enzymes was an effective method for TPS extraction and could significantly improve TPS’s free radical scavenging activity [28]. Chang et al., used pectinase-assisted extraction to obtain green tea TPS, and the primary extraction process was as follows: the ground tea powder was heated in a water bath at 90 °C for 2–4 h, 0.5% pectinase (260,001 PGU/mL, *v*/*w*) was added and incubated at 40 °C for 30 min, and then the enzyme was inactivated by heating at 90 °C for 1 h. The prepared TPS presented excellent immune stimulation and protection against immune cells [30]. In addition to bioactivity, yield is also a concern for enzymolysis extraction. Zhu et al., used mixed enzymes (cellulase:pectinase:glucanase = 1:1:2) for crude green tea polysaccharide (CTPS) extraction at 50 °C (30 min), followed by boiling to inactivate the enzyme (10 min) and extracting in a water bath at 50 °C for 80 min. The whole process was repeated three times. The CTPS obtained with this method had a high total sugar content (71.83%), which could mainly be attributed to the gentle and efficient destruction of the cell walls by mixed enzymes [21,44]. Wang et al., used a 0.5% (m/v) pentosan complex enzyme solution (45 °C, pH 5.5) to extract TPS from green tea leaves and flowers pretreated with 95% ethanol for 2 h. After filtration, the same extraction process at the same temperature was repeated. The yields of two TPSs obtained with this method were 4.08% and 6.88%, respectively, which were much higher than those obtained with hot water extraction under the same conditions (1.28% and 2.93%, respectively) [38]. Compared to the conventional solvent extraction method, the enzymolysis extraction method has the advantages of a high extraction efficiency, strong specificity, and high extraction rate. In addition, it can reduce the environmental pollution caused by using a large amount of solvent and thus has broad application prospects. However, since the price of the enzyme is relatively high and its activity is affected by various factors, the extraction conditions for enzymolysis extraction must be strictly controlled to effectively obtain a higher extraction rate.

### 2.5. Other Extraction Methods

Some new methods for TPS extraction in addition to the above-mentioned common extraction methods have also been reported. For example, Xu et al., optimized extraction conditions using a hydro/solvothermal method. They used high temperature and pressure (120 °C, 0.1 MPa) to infiltrate water into the tea leaves of Zhongcha 108 to destroy the cell structure, thereby separating TPS [1]. The extraction rate of crude polysaccharides obtained with this method was 4.7%, which was much higher than that of TPS obtained with ordinary hot water extraction, such as Ziyang green tea (3.46%) [22], Huangshan Maofeng tea (2.3%) [23], and Keemun black tea (3.2%) [24]. Sun et al., used alkali-assisted extraction to extract Fuzhuan brick tea polysaccharide (FBTPS); the extraction conditions were a 60 °C extraction temperature and a 0.1 mol/L NaOH solution (pH = 10.0). Compared to hot water extraction, the yield of FBTPS by alkaline extraction was found to have a greater impact on the monosaccharide composition and yield [30]. In addition, emerging extraction technology supercritical fluid extraction (SFE) has also been used to extract polysaccharides in recent years. Many researchers have used SFE to extract various plant-derived polysaccharides, though there are still few applications of this process for TPS extraction. Chen et al., extracted TPS with a CO_2_-based SFE method, and they determined the optimum parameters of this method in TPS extraction as a particle size of 380 μm, 20% absolute ethanol, an extraction pressure of 35 MPa, an extraction temperature of 45 °C, an extraction time of 2 h, which enabled a TPS extraction rate of up to 92.5%. Moreover, the TPS obtained with this method was significantly bioactive [31]. Although the SFE method is impressive, manageable, efficient, and environmentally-friendly, it is still not as common as other extraction methods in practical applications due to its expensive and time-consuming equipment. In addition, Li et al., found that extraction via a anionic reverse micelle system exhibited the advantages of a fast mass transfer, high selectivity, and low cost [32]. In short, various auxiliary methods for TPS extraction are able to improve the bioactivity of polysaccharides, shorten extraction times, and improve extraction yields.

## 3. Preliminary Physicochemical Properties of TPS

### 3.1. Monosaccharide Composition

The monosaccharide composition of TPS is usually analyzed using gas chromatography (GC) and GC mass spectrometry (GC–MS) after the hydrolysis of glycosidic bonds with trifluoroacetic acid and derivatization with acetic anhydride [45]. It has been reported that TPS is formed by linking 2–10 monosaccharides in different arrangements with glycosidic bonds (Table 2). Zhu et al., detected and compared the monosaccharide compositions of CTPS obtained with four different extraction methods (WE, UAE, MAE, and EE) [21]. The results showed that the monosaccharide compositions of the four CTPS were the same. They all contained rhamnose (Rha), arabinose (Ara), galactose (Gal), glucose (Glc), xylose (Xyl), mannose (Man), fucose (Fuc), and galacturonic acid (GalA); the molar ratio of Glc was the highest at 29.22%, 36.05%, 31.09%, and 44.24%, respectively, for WE, UAE, MAE, and EE. These results were the same as those of Wang et al., [46], indicating that although different extraction techniques can affect the composition of monosaccharides, Glc in CTPS may be the main monosaccharide component [21]. Zhu et al., also obtained two homogeneous TPSs (ASe-TPS2 and NSe-TPS2) from natural selenium-enriched and artificial selenium-enriched green teas, respectively, with uronic acid contents as high as 65.45% and 69.98%, respectively, confirming that they were typical acidic polysaccharides [47]. Further monosaccharide composition analysis by ion chromatography (IC) showed that ASe-TPS2 mainly contained Rha, Ara, Glc, Xyl, and GalA with a molar ratio of 1.93: 7.05: 1.00: 1.05: 26.12, respectively, while NSe-TPS2 was mainly composed of Ara, Gal, glucuronic acid (GlcA), and GalA at a molar ratio of 0.59: 1.00: 0.49: 1.24, respectively. These results suggest that different selenium-enriched methods may also lead to differences in the monosaccharide composition of TPS, and differences in uronic acid content may affect its chemical properties or bioactivity. Wang et al., also used the IC method to detect the purified components of selenium-enriched green tea polysaccharides (Se-TPS1, Se-TPS2, and Se-TPS3). They found that the three purified components were also acidic polysaccharides [48], and though their monosaccharide composition was the same as that of NSe-TPS2, their molar ratios were different. Yang et al., extracted crude tea polysaccharide (CTPS) and two fractions, TPS-1 and TPS-2, from Qingzhuan brick tea. TPS-2, with the lowest uronic acid content (24.45 mg/g), showed stronger ferric ion-reducing antioxidant power (FRAP) and in vitro scavenging capability against 1,1-diphenyl-2-picrylhydrazyl (DPPH) and 2,2-azino-bis(3-ethylbenzothiazoline-6-sulphonic acid) ABTS radicals [49]. According to related reports, a large amount of uronic acid in polysaccharides may lead to a stronger ABTS free radical scavenging capability [50], whereas the monosaccharide composition of TPS may have a huge impact on its FRAP properties [51]. The crude Fuzhuan brick tea polysaccharide (FBTPS) obtained with the hot water extraction method (1:10, *w*/*v*; 70 °C) by Chen et al., contained 37.78% uronic acid content, and its monosaccharide composition comprised D-ribose (Rib) (1.69 mol%), Man (3.66 mol%), Ara (11.83 mol%), Rha (12.11 mol%), Gal (19.15 mol%), Glc (21.97 mol%), GlcA (1.41 mol%), and GalA (28.17 mol%) [52]. Wang et al., further purified FBTPS and found that FBTPS-3 was the main component of FBTPS (the yield was 37.7%), and its monosaccharide composition included Man, Rha, GalA, Gal, and Ara at a molar ratio of 8.7: 15.5: 42.2: 19.7: 13.9, respectively [53]. Among them, the high GalA composition of FBTPS-3 corresponded to its high uronic acid content (40.4%), indicating that FBTPS-3 is an acidic polysaccharide. Ke et al., obtained crude green tea polysaccharide (CGPS) by extraction with boiling water at 100 °C and then further purified it to obtain homogeneous GTP consisting only of Glc [54]. Li et al., also used boiling water to extract Yingshan Cloud Mist green tea polysaccharide (GTPS) [55], a neutral polysaccharide composed of Rha, Ara, Xyl, Man, Glc, and Gal at a molar ratio of 11.4: 26.1: 1.9: 3.0: 30.7: 26.8, respectively. Although there was no uronic acid in the studied GTPS, it also had certain in vitro anti-radical activity, which may have been related to its high Glc and Gal contents. Gu et al., isolated and purified two selenium-enriched polysaccharides, SeTPS-1 and SeTPS-2, from green tea crude leaves by extraction at high temperature and high pressure (150 °C, 6 MPa) [56]. Component content and monosaccharide detection showed that their selenium contents were 23.50 μg/g and 13.47 μg/g, respectively. SeTPS-1 did not contain any uronic acid, and its monosaccharide composition mainly comprised Glc and Gal with a molar ratio of 80.1:2.3, respectively. The uronic acid content in SeTPS-2 was found to be 15.77%, and its monosaccharide composition mainly comprised Glc and Gal with a molar ratio of 80.1:2.3, respectively. Importantly, SeTPS-2 had stronger antioxidant capacity in vitro, which may have been related to its rich uronic acid content. Wang et al., analyzed the monosaccharide composition of yellow tea polysaccharide (YTPS) before and after sonication by HPLC and found that sonication did not change its monosaccharide composition but did have a slight effect on the molar ratio. Both YTPSs mainly consisted of Rha, with small amounts of Man, Rib, GlcA, Gal, and Ara [26]. Chen et al., explored the effect of an ultra-high pressure (200–600 MPa, 25 °C) treatment on the monosaccharide composition of large-leaf yellow tea polysaccharide (LYTP) [57]. LYTP was mainly composed of Ara, Gal, GalA, Rha, Glc, GlcA, and Man. After ultra-high pressure treatment, the content of GlcA in LYTP significantly increased and the contents of Ara, Gal, and GlcA significantly decreased. The shear force generated by ultra-high pressure was able to break the glycosidic bonds connecting Ara, Gal, and GlcA in the main chain or side chain, thereby promoting TPS degradation. However, fragments linked by a large amount of GalA were more stable, thus increasing the proportion of GalA [58]. In addition, numerous studies have shown that acidic polysaccharides generally have high bioactivity [59,60].

### 3.2. Molecular Weight (Mw)

Mw is one of the most important physical properties of polysaccharides. Numerous studies have shown that Mw is not only an essential indicator for judging the chemical properties of polysaccharides but may also affect their bioactivity. The Mw size of TPS is closely related to the type of tea and the purification process [61,62]. In most studies, gel permeation chromatography (GPC), gel filtration chromatography (GFC), and multi-angle laser light scattering (MLLS) detection methods have been employed to determine the Mw of TPS [45]. Zhu et al., used high-performance gel permeation chromatography (HPGPC) to determine the Mw of crude TPS obtained with four different extraction methods (WE, UAE, MAE, and EE) [21]. The results showed that the Mw of WE-CTPS was mainly distributed at 2558 kDa, accounting for 59.46% of the area. However, the Mw distribution curves of UAY-CTPS and EE-CTPS shifted to the right, the number of peaks at 1000 kDa and 3000 Da significantly increased, and the Mw of UAE-CTPS was smaller than that of EE-CTPS. In addition, MAE treatment resulted in the vigorous degradation of small-Mw TPS but had little effect on high-Mw TPS chains. MAE treatment will damage the cell structure and accelerate the collision between small molecules, easily leading to the fragmentation of small-Mw polysaccharides [63]. In addition, polysaccharides with smaller Mw values may find it relatively easier to enter the cell interior to escape the stress of the immune system, thus showing better bioactivity than large-Mw polysaccharides [42]. Another study by Zhu et al., also proved this conclusion by detecting the in vitro inhibitory activities of α-glucosidase and α-amylase on four CTPSs [21]. In their other study, it was found that different selenization methods also affected the Mw of Se-TPS. Among them, the Se-TPS obtained with the natural Se-enriched method had a higher Mw of 244.32 kDa than that of NSe-TPS2 (6.73 kDa) [47]. The Mw of three purified selenium-enriched green tea polysaccharide fractions (Se-TPS1, Se-TPS2, and Se-TPS3) obtained under WE (70 °C) were tested by Wang et al., Under WE at 90 °C, Se-TPS1 and Se-TPS2 (as homogeneous polysaccharides,) had lower Mw values of 110 kDa and 240 kDa, respectively, compared to NSe-TPS2. At the same time, Se-TPS3 was found to be a polysaccharide polymer with an Mw range of 250–920 kDa [48]. Chen et al., conducted a study on the digestion of TPS in the gastrointestinal tract, and they found that the initial Mw of FBTPS was 828 × 10^3^ g/mol and that the Mw of FBTPS did not change after the “digestion” treatment of in vitro digestive juice. However, after being acted on by microorganisms in the large intestine, FBTPS’s Mw decreased with the prolongation of treatment time [52]. Wang et al., found similar results when exploring the purified components of FBTPS with an Mw of 741 kDa [53]. Li et al., obtained GTPS with an Mw of 96.9 kDa by boiling water extraction, and they found that GTPS showed better in vitro antioxidant activities in a dose-dependent manner [55]. Previous studies have shown that polysaccharides’ antioxidant activity is related to their Mw and that the Mw is mainly distributed between 10 and 1000 kDa [64]. Sun et al., found various antioxidant activities of green tea TPSs (TPS1, TPS2, and TPS3) with different Mw values (8.16, 4.82, and 2.31 kDa, respectively); TPS2, with a medium Mw, had the strongest hydroxyl-radical, ABTS free radical, and hydroxyl-radical scavenging capabilities [65]. Compared to those with smaller Mw values, large-Mw TPSs were found to have tighter spatial structures, resulting in fewer active groups being exposed to the outside and weakening their capacity to terminate free radical chain reactions [66,67]. Zhao et al., obtained similar results and found that the cellular repair capacity of TPSs was positively correlated with their antioxidant activity [67]. Gu et al., separated and purified two Se-enriched polysaccharides, SeTPS-1 and SeTPS-2, under high-temperature and high-pressure conditions, resulting in Mw values of 17 and 13 kDa, respectively. SeTPS-2 had a stronger antioxidant capacity in vitro, which may have been related to its lower Mw content [68]. By sonicating YTPS, Wang et al., found that the Mw of YTPS-3 purified with 30% ethanol decreased from 37.7 to 15.1 kDa, proving that ultrasonic irradiation promotes the fragmentation of polysaccharides and results in a decrease in Mw [69]. The YTPS after ultrasound showed more substantial antioxidant capacity than pre-ultrasound YTPS, further confirming that a low Mw could promote antioxidant capacity, which may be attributed to the larger surface area and number of reaction sites of degraded YTPS [26]. Chen et al., found that UHP treatment significantly reduced the Mw of large leaf yellow tea TPS [70], and turbulent flow and high shear forces formed during UHP processing may cause cell deformation or even rupture and lead to fragmentation and degradation of polysaccharides [57,71].

### 3.3. Solubility

Since TPS contains a large number of polar groups [47,72], it has a strong affinity for water molecules, which allows it to restrict the flow of water. The hydrophilicity of TPS is related to its Mw. The smaller-Mw and less-branched chains of TPS have higher water solubility. Usually, proper heating would promote the dissolution of polysaccharides. Zhu et al., compared the solubility of coarse green tea polysaccharide (CTPS) obtained with different extraction methods. They found that the time for a complete dissolution of CTPS decreased with increasing temperature. At the same temperature, the dissolution time of CTPS obtained with hot water extraction was always the longest [21]. In addition, they also investigated the solubility of TPSs (DTPS-1, DTPS-2, DTPS-3, DTPS-4, DTPS-5, and DTPS-6) from dark tea, and the time for the complete dissolution of DTPS was also negatively correlated with the heating time. When the temperature exceeded 80 °C, the solubility of different DTPSs was almost the same. Additionally, at the same temperature, the dissolution time of DTPS-3 was always the shortest, probably because its structure was more fragmented and then increased the surface area for its reaction [10].

### 3.4. Viscosity

Due to the particularity of the solubility of polysaccharides, polysaccharides have high viscosity in aqueous solutions and even form gels [73]. The principle is that polysaccharide molecules exist in the form of random coils in solution, and their tightness is related to the composition of monosaccharides and their connection form [74]. When polysaccharide molecules are stirred and rotated in the solution, they need to occupy a large space. At the same time, the collision probability between polysaccharide molecules is elevated and the friction force is enhanced, thus generating a higher viscosity [74]. Due to their specific structural compositions, different polysaccharides produce a high viscosity even at low concentrations. Generally, the viscosity of polysaccharide molecules is not only related to the composition and connection form of monosaccharides but also their Mw. Wang et al., performed intrinsic viscosity analysis on four purified fractions of oolong tea polysaccharides (OTPSs) with different Mw values: OTPS1 (>80 kDa), OTPS2 (30–80 kDa), OTPS3 (10–30 kDa), and OTPS4 (<10 kDa) [75]. The intrinsic viscosities of the four OTPS components were 239.56, 162.63, 7.75, and 2.57 mL/g, respectively, which indicated that their intrinsic viscosities increased with relative decreases in Mw. Xu et al., isolated and purified six TPS components with different Mw values from green, oolong, and black teas, and then they performed an intrinsic viscosity analysis [76]. The Mw values of GTPS1, OTPS1, and BTPS1 were less than 80 kDa, while the Mw values of GTPS2, OTPS2, and BTPS2 were greater than 80 kDa. Their intrinsic viscosities were 53.96 mL/g, 60.28, 60.29, 106.95, 106.76, and 104.67 mL/g, respectively. The intrinsic viscosity of TPS1s was found to be lower than that of TPS2s, which may have been related to the decrease in Mw.

### 3.5. Emulsifying and Stability

Emulsifiers are a crucial material in the production of food, cosmetics, and pharmaceuticals. Emulsifiers contain hydrophilic and hydrophobic regions and are rapidly adsorbed at the oil–water interface, stabilizing emulsion through steric hindrance or electrostatic interactions [77]. Currently, the most commonly used emulsifiers are mainly chemically synthesized ones, such as fatty acid monoglycerides, Tween-80, and sucrose esters [78]. With consumers’ increasing pursuit of “green products and healthy life”, the development of natural emulsifiers has received great attention [79]. Polysaccharide-based emulsifiers are some of the most commonly used natural emulsifiers in the food industry [49]. The emulsification of polysaccharides improves with increases in solution viscosity, but viscosity is not the main factor affecting emulsification [80]. The Mw of polysaccharides is related to the viscosity, interfacial activity, and hydrophobic groups of polysaccharide solutions. Thus, the Mw of polysaccharides may also affect their emulsification. Studies have shown that increases in Mw can increase polysaccharides’ steric hindrance, prevent droplets’ aggregation and flocculation, and improve emulsification [81]. The effects of chemical modification on the Mw of polysaccharides can be divided into two types: when the alkyl chain is short and the degree of substitution is low, the degradation of polysaccharides is dominant and the Mw is reduced; when the alkyl chain is long and the degree of substitution is high, the Mw and hydrophobicity of the polysaccharide are increased, resulting in a reduction in the particle size of the emulsion and an improvement of the emulsification [82]. The Mw of acetylated pectin polysaccharides was found to first decrease and then increase with the rise in substitution degree, and the viscosity was found to first decrease and then increase with the rise in substitution degree [83]. At this time, the emulsifying properties are improved, so the degree of substitution can increase the emulsifying properties of the polysaccharide. The stability of an emulsified polysaccharide solution is also closely related to the structure of the polysaccharide molecule itself. Uncharged linear polysaccharides can be combined with hydrogen bonds after forming a colloidal solution. With extensions of time, the degree of association becomes stronger and precipitation or molecular crystallization will occur under the action of gravity. Branched polysaccharide colloids also become unstable due to molecular aggregation, though at a slower rate. In addition, charged polysaccharide colloids have higher stability due to the repulsion of the same charge between molecules. In addition, the protein–polysaccharide conjugate formed by the distribution of the protein part along the polysaccharide chain combines the characteristics of proteins and polysaccharides, which is also beneficial to improving emulsification and stability to a certain extent [84]. In a report by Chen et al., an alkali-extracted tea polysaccharide conjugate (TPC-A) was used to stabilize oil-in-water emulsions, and TPC-A was shown to have a favorable protective effect on catechins and could be used as a natural emulsifier [85]. Li et al., also obtained a TPS conjugate with good emulsifying properties and excellent antioxidant activity from Chin brick tea [86]. The effects of a certain degree of heat treatment on the physicochemical and functional properties of TPC were also investigated. The heat treatment of TPC (TPC-3d) at 110 °C for three days significantly improved its emulsification activity and stability but did not affect its antioxidant activity [45]. Chen et al., studied the emulsifying properties and emulsifying stability of polysaccharide conjugates (TPC-C) from Chin-Brick Tea. They found that TPC-C had no effect on the dynamic interfacial tension and particle size formation of emulsions or storage, and it showed excellent potential as a natural emulsifier in terms of stability; the TPS part mainly provided pH stability for the TPC-C stabilized emulsion [87]. In another study, Chen et al., extracted a TPS conjugate (gTPC) from low-grade green tea and obtained two purified components: gTPC-1 and gTPC-2 [88]. The high-Mw gTPC-1 fraction exhibited a higher emulsion stability than the low-Mw gTPC-2, which may be attributed to differences in polysaccharide chain length and conformation in the conjugates. Compared to the lower-Mw polysaccharides, higher-Mw polysaccharides were able to more efficiently coat the droplet surface. At the same time, the high-Mw polysaccharide conjugates improved the emulsion’s salt and thermal stability, which may have been due to the capability of high-Mw polysaccharides to form thicker charged coatings, causing an increased steric hindrance between droplets and a greater electrostatic repulsion compared to low-Mw polysaccharides [89,90].

## 4. In Vitro Bioactivity of TPS

**Figure 2:**
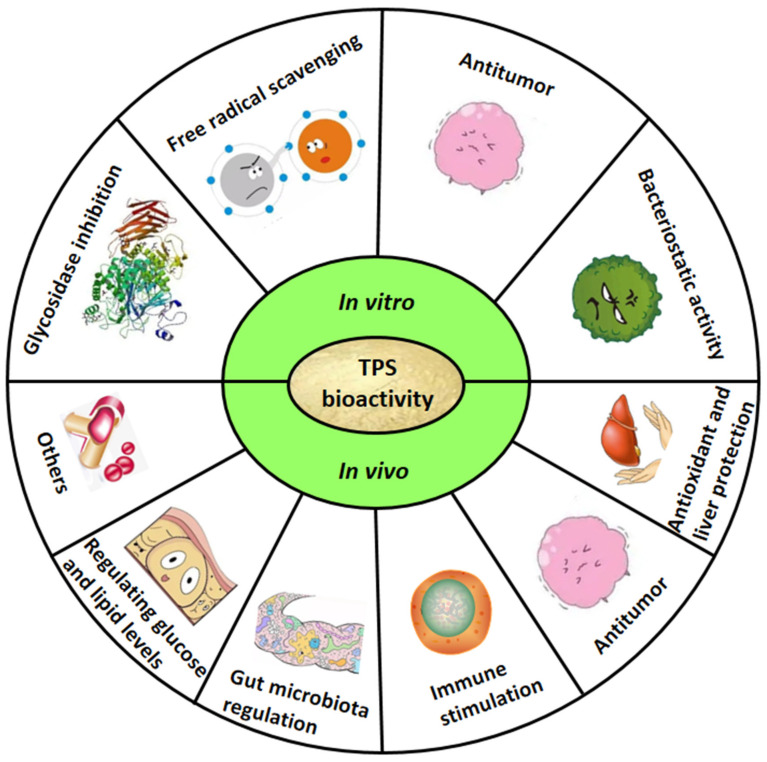
In vitro and in vivo bioactivities of TPS.

As a bioactive polysaccharide, TPS has been reported to show favorable performance in various in vitro activity evaluation models (Figure 2).

### 4.1. Glycosidase Inhibition

Alpha-glucosidase is an essential enzyme in carbohydrate digestion. In a regular diet, starch is first cleaved into oligosaccharides by α-amylase. The oligosaccharides are hydrolyzed by α-glucosidase to release glucose, which is absorbed into the blood by intestinal epithelial cells. Thus, the inhibition of α-glucosidase, an enzyme that has been proposed as a therapeutic target for regulating postprandial hyperglycemia, prevents excess glucose absorption in the small intestine and controls postprandial hyperglycemia [91]. Acarbose can inhibit alpha-glucosidase and prevent elevated postprandial blood glucose levels, and it is widely used in treating patients with type 2 diabetes. However, synthetic chemicals have various side effects, such as flatulence and diarrhea [92]. Therefore, natural α-glucosidase inhibitors without side effects have attracted more and more attention. Chen et al., isolated three TPS fractions—GTPS, OTPS, and BTPS—from green, oolong, and black tea, respectively, and then compared their in vitro α-glucosidase inhibitory activities [35]. Among them, BTPS could inhibit α-glucosidase activity in dose-dependent manner (14.3–91% with an increase from 25 to 200 μg/mL), whereas the inhibitory activities of GTPS and OTPS on α-glucosidase were lower. This may have been due to differences in the conformation and composition of polysaccharides, which lead to differential interactions between aminoglycosides and the hydrophobic pockets of enzymes. Xu et al., prepared crude TPSs of GTPS, OTPS and BTPS according to the same method as above, and they further performed ultrafiltration to obtain six TPS components: GTPS1 (<80 kDa), GTPS2 (>80 kDa), OTPS1 (<80 kDa), OTPS2 (>80 kDa), BTPS1 (<80 kDa), and BTPS2 (>80 kDa). BTPS1, BTPS2, OTPS1, and OTPS2 were found to have a dose-dependent inhibitory effect on α-glucosidase activity, and BTPS1 exhibited the most robust α-glucosidase inhibitory activity. Additionally, the inhibitory activities of GTPS1 and GTPS2 against α-glucosidase were weaker than those of other TPS fractions, results consistent with those of Chen et al., In addition to the type of tea, the degree of fermentation of tea can also affect its glycosidase inhibitory activity. The aging (light fermentation) process was found to significantly enhance the antioxidant and α-glucosidase inhibitory activities of Pu-erh TPS [76]. Xu et al., extracted three TPSs (PTPS-1, PTPS-3, and PTPS-5) from Pu-erh tea with different fermentation years (first, third, and fifth) and then tested their α-glucosidase inhibitory activity [36]. Based on EC50 (concentration for 50% of maximal effect), PTPS-5 inhibited α-glucosidase at least 3 times more than the positive drug (acarbose), PTPS-3 inhibited α-glucosidase comparably to acarbose, and the inhibition of PTPS-1 was the weakest. These results suggest that the aging time of Pu-erh tea TPSs may be positively correlated with the inhibitory effect of PTPS on α-glucosidase. Notably, processing technology also plays a significant role in the inhibition of glycosidase activity by TPS. Wei et al., investigated the effects of different extraction methods, including WE, UAE, and MAE, on the bioactivity of TFPS [93]. TFPS extracted with the UAE and MAE methods had almost no inhibitory activity on β-glucosidase, while the TFPS extracted with the TWE method had little inhibitory activity on β-glucosidase. The inhibitory activity of glucosidase (83.3% inhibition rate) for TFBS extracted with WE was significantly higher than that of TPS from tea leaves. At the same time, Wang et al., compared the effects of different drying methods on the bioactivity of TPS. They found that the TPS obtained with freeze drying (TPS-F) had 92.8% and 82.75% inhibition rates of α-amylase and α-glucosidase, respectively. Its inhibitory activity was significantly more substantial than that of TPS obtained with vacuum drying (TPS-V) and spray drying (TPS-S) [94]. In addition, the species of TPS may also affect its glycosidase inhibitory activity. Wang et al., isolated the acidic TPS fraction (TP-1) from Maofeng tea via acid extraction, and its inhibitory activities (IC50, the half-maximal inhibitory concentration) against α-glucosidase and α-amylase were 394.3 and 90.1 µg/mL, respectively [95]. Recently, a study by Zhu et al., showed that the inhibition of α-glucosidase and α-amylase activities by coarse green tea TPS might be related to the Mw of TPS [21]. They pointed out that the Mw of TPS was negatively correlated with the inhibition of glycosidase activity. Accordingly, they also found that the inhibitory activity of dark tea TPS on glycosidase may have a significant positive correlation with its uronic acid content [10]. Further reports by Fan et al., showed that higher purities of TPS led to weaker inhibitory activities on α-glucosidase and α-amylase [96].

### 4.2. Free Radical Scavenging

Oxidative stress caused by the transition of oxygen-derived free radicals is an important cause of the occurrence and development of many diseases, such as cancer, hypoglycemia, atherosclerosis, and rheumatoid arthritis, as well as degenerative diseases related to aging [97]. The free radical scavenging activity of TPS has also been widely reported. Chen et al., compared the in vitro free radical inhibitory activities of GTPS, OTPS, and BTPS from green, oolong, and black teas [35]. They found that the three TPSs showed significant antioxidant activities on DPPH free radicals, hydroxyl radicals, and lipid peroxidation (*p* < 0.05). Among them, the scavenging effects of GTPS and BTPS were better than that of OTPS, and GTPS showed the strongest lipid peroxidation inhibitory activity (IC50 = 75 μg/mL). Sun et al., extracted a water-soluble polysaccharide (KBTP) from Keemun black tea and evaluated its in vitro antioxidant capacity. The KBTP showed strong DPPH free radical scavenging, superoxide anion-radical scavenging, and iron-reduction capacity in a dose-dependent manner, though with lower values than the positive control benzyl alcohol (BHT) [24]. Xiao et al., investigated and compared the DPPH free radical scavenging activity of four corresponding crude tea polysaccharides—XTPS, TTPS, CTPS, and HTPS—prepared from four types of expired tea leaves on the market—Xihu Longjing, Huizhoulvcha, Chawentianxia, and Anxi Tieguanyin, respectively [13]. The scavenging activities of the four TPSs against DPPH showed a similar effect. In the concentration range of 25–200 g/mL, the scavenging effects of the four TPSs were enhanced with the increase in the concentration, but all were lower than that of Vc. Among them, the content of polyphenols in CTPS was relatively low (6.53%), but its DPPH free radical scavenging activity was similar to that of TTPS, indicating that the main antioxidants in CTPS are polysaccharides. Xu et al., prepared crude tea flower polysaccharides (TFPS) and obtained three purified components: TFPS-1, TFPS-2, and TFPS-3 [98]. In vitro antioxidant testing revealed that all TFPS samples had appreciable scavenging activities of DPPH, superoxide anion, and superoxide anion free radicals in a concentration-dependent manner. Among them, TFPS-1 had the strongest in vitro antioxidant capacity. Xu et al., compared the free radical scavenging capability of six TPS samples (GTPS1, GTPS2, OTPS1, OTPS2, BTPS1, and BTPS2), and they found that the fermentation process and the Mw of TPS had significant impacts on the DPPH free radical scavenging [76]. Another study by Xu et al., indicated that PTPS-5, with the highest studied proportion of low-Mw polysaccharides, had the strongest free radical scavenging capability [99]. Likewise, Sun et al., showed that a green tea TPS fraction with a moderate Mw exhibited the strongest in vitro free radical scavenging activity and reducing power of studied fractions [65]. Thus, the Mw of TPS may play an important role in its antioxidant activity, and low-Mw TPSs generally exhibit higher radical scavenging capacities [97]. In addition, the hydroxyl groups in polysaccharides are also an important factor affecting their free radical scavenging activity [100]. Other studies have shown that the content of uronic acid is closely related to the antioxidant activity of TPS [10,101]. In addition, changes in the spatial structure of TPS caused by ultrasound can also enhance its scavenging activity to DPPH, superoxide, and hydroxyl radicals [26].

### 4.3. Antitumor Activity

In recent years, TPS has received extensive attention due to its broad therapeutic properties and relatively low toxicity to normal cells, and it is expected to become an alternative or adjunct to traditional anticancer drugs [102]. Liu et al., isolated and purified a water-soluble homogeneous polysaccharide (DTP-1) from dark brick tea, and then they evaluated the cytotoxic activity of DTP-1 on cancer cells and normal cells in vitro [103]. The results showed that DTP-1 had significant in vitro anti-tumor effects, especially on A549 and SMMC7721 cells, and the inhibitory effect of DTP-1 on cancer cell proliferation was positively correlated with its dose. In addition, DTP-1 could effectively inhibit the proliferation of cancer cells, induce apoptosis, and inhibit migration. At the same time, it hardly affected the growth and viability of normal cells. Wang et al., isolated a selenium-enriched TPS (Se-ZYTP) from Ziyang selenium-enriched green tea and investigated its in vitro antitumor activity against human osteosarcoma cells (U-2 OS). Both MTT and lactate dehydrogenase (LTH) assays demonstrated that Se-ZYTP could significantly inhibit the proliferation of U-2 OS cells in a concentration-dependent manner [104]. Zhou et al., extracted a green tea polysaccharide (GTPS) from crude green tea and tested its inhibitory effect on the viability of colon cancer cells (CT26). The results showed that the inhibitory effect of GTPS on CT26 cells was concentration-dependent and time-dependent. At the highest concentration (800 μg/mL), the anticancer effect of GTPS was stronger than that of the positive control Lentinus edodes (LNT). Additionally, GTPS was not toxic to normal rat intestinal epithelial cells. This study also showed that GTPS intervention significantly up-regulated signal transduction pathways related to apoptosis, lysosomes, mitochondria, and cell death, suggesting that GTPS might target lysosomes and activate caspase −9/−3-inducing apoptosis of CT26 cells, thereby exerting anti-cancer effects [105]. In addition, Xu et al., conducted an anticancer evaluation on tea flower polysaccharide (TFPS) and its purified fractions (TFPS-1, TFPS-2, and TFPS-3). They found that these samples could significantly inhibit human gastric cancer cells (BGC-823) in a concentration- and time-dependent manner. Among them, TFPS-1 and TFPS-3 exhibited higher in vitro antitumor activities than TFPS-2, possibly due to their differences in monosaccharide composition, sulfate content, and antioxidant activity [98]. Yang et al., investigated the effect and possible mechanism of green tea polysaccharide (GTP) in anti-prostate cancer (PC). They found that GTP could promote PC cell apoptosis by increasing Bcl2-associated X protein (Bax)/B-cell lymphoma-2 (Bcl-2) ratio and caspase-3 protein expression while decreasing *micro RNA-93* (*miR-93)* expression. Of these, *miR-93* might be a key target of GTP in PC treatment [54].

### 4.4. Bacteriostatic Activity

The human body has a diverse microflora that contain more cells than the body, and these microbiotas are mainly composed of bacteria. The ordinary microbial communities in the respiratory tract, gastrointestinal tract, and skin interact with the human body’s innate immunity. These microbial communities also produce metabolites that serve as essential nutrients for human cells and play a protective role by inhibiting pathogenic bacteria [106]. There is growing evidence that interactions between humans and their symbiotic flora may play an essential role in human health. However, bacterial attachment to human epithelial cells may also lead to skin infections, inflammation, and some pathogenic diseases [107]. For example, *Helicobacter pylori* is a micro-aerophilic Gram-negative bacterium that exclusively colonizes the gastric mucosa of humans and primates, causing chronic active or type B gastritis, duodenal ulcers, gastric cancer, and mucosal-associated lymphoid tumors [108]. *Propionibacterium acnes* (*P. acnes*) is an anaerobic Gram-positive bacterium that is common in microscopic studies of adult skin and predisposes one to the formation of human cutaneous acne [109]. *Staphylococcus aureus* is an aerobic Gram-positive bacteria that mainly hides on the skin and mucous membrane surfaces, resulting in atopic dermatitis [110]. In addition to being a healthy drink, tea has been regarded as an anti-cancer and antibacterial herb since ancient times. As an essential active ingredient in tea, TPS has also been reported to show favorable antibacterial activity. Lee et al., obtained an acidic TPS (ATPS) from green tea and analyzed its anti-adhesion effect against pathogenic bacteria (*Staphylococcus aureus*, *P. acnes*, and *Helicobacter pylori*) [111]. The results showed that ATPS had a significant inhibitory effect on pathogenic bacteria-mediated hemagglutination, with a minimum inhibitory concentration of between 0.01 and 0.1 mg/L. In addition, ATPs had no inhibitory effect on beneficial commensal bacteria such as *Staphylococcus epidermidis*, *Escherichia coli*, and *Lactobacillus acidophilus*, suggesting that ATPs could be applied as selective natural antiadhesive polymers to some pathogenic bacteria. However, there have been few reports on the significant bacteriostatic activity of TPS, and more studies are needed to support this evidence of this property.

## 5. In Vivo Bioactivity of TPS

**Figure 3:**
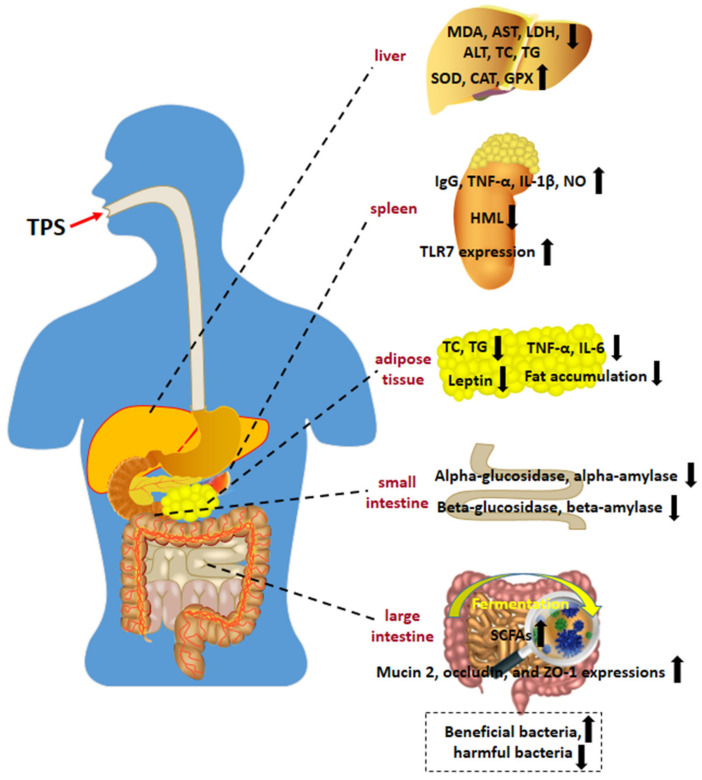
A potential mechanism by which TPS exerts its in vivo bioactivity.

In addition to the presented in vitro bioactivities, TPS also showed favorable in vivo bioactivities (Table 3 and Figure 2), and its potential regulatory mechanism is presented in Figure 3.

### 5.1. Antioxidant and Hepatoprotective Activity

Oxidative stress has been reported to be associated with many diseases, such as cancer, Alzheimer’s disease, nephritis, arteriosclerosis, and diabetes, due to the overproduction of oxygen free radicals [4]. The liver is a vital detoxification organ in the human body and plays a key role in the metabolism of various endogenous and exogenous harmful substances [3]. Many in vivo studies in mouse models for the antioxidant and liver-protective effects of TPS have been reported. Ren et al., fed healthy male Kunming mice with 20% fructose water for eight weeks and then provided different concentrations of Ziyang selenium-enriched green TPS (Se-TPS) for eight weeks. Se-TPS was shown to be able to significantly attenuate hepatic steatosis and oxidative stress injury in mice [112], and Se-TPS could substantially attenuate hepatic steatosis and oxidative stress injury in mice [112]. Wang et al., explored the hepatoprotective effect of Ziyang tea polysaccharide (ZTPS) on CCl_4_-induced oxidative liver injury in mice [113]. They found that the administration of ZTPS (100, 200, and 400 mg/kg) to mice prior to CCl_4_ treatment significantly inhibited CCl_4_-induced elevations in liver MDA levels, AST, lactate dehydrogenase (LDH), and serum ALT. In addition, ZTPS-treated mice exhibited regular SOD and glutathione peroxidase (GPx) activities compared to a CCl_4_-induced group. Sun et al., studied the antioxidant activity of TPS in exhaustive exercise mice. They found that MDA levels in the heart, liver, and plasma were reduced after 30 days of TPS treatment compared to a control group [131]. Lu et al., investigated the antioxidant and hepatoprotective effects of Huangshan Maofeng acid polysaccharide (HMTPS) on CCl_4_-induced oxidative liver injury in mice [23]. The results showed that HMTPS could significantly regulate the serum markers of triglyceride (TG), total cholesterol (TC), aspartate aminotransferase (AST), and alanine aminotransferase (ALT) in mice with liver injury induced by CCl_4_. In addition, HMTPS could significantly increase the antioxidant levels of SOD and hepatic glutathione and could reduce the formation of the hepatic lipid peroxidation products 15-F2t isoprostanes and MDA. Similarly, a study by Xu et al., indicated that TFPS could significantly reduce the serum levels of ALT and AST in mice with CCl_4_-induced hepatotoxicity in a dose-dependent manner while considerably reducing the level of MDA in the liver and enhancing antioxidant enzyme activity (SOD and GPx) [98]. Sun et al., reported that Keemun black tea polysaccharide (KBTP) intervention in mice prior to CCl_4_ injection significantly prevented CCl_4_-induced elevations in serum ALT, AST, TG, TC, and MDA levels [24]. Compared to CCl_4_-induced liver injury mice, KBTP-pretreated mice exhibited better liver body indexes and higher GSH and SOD activities. Accumulating evidence has revealed that TPS can reduce oxidative stress in the body and may be a potent antioxidant in medicines and functional foods. However, some researchers hold the opposite view on the antioxidant activity of TPS. Wang et al., separated TPS components from a crude TPS extract and then compared their DPPH free radical scavenging, reducing power, and hydroxyl radical scavenging activities. They found that the purified TPS had almost no antioxidant activity and that the primary antioxidants in crude TPS may be polyphenols [132]. Importantly, single TPS fractions should be isolated and purified to test their antioxidant and hepatoprotective effects individually, which would help clarify TPS’s main antioxidant and hepatoprotective components and help improve TPS preparation.

### 5.2. Antitumor Activity

In cellular models, TPS has been reported to play an essential role in tumor and cancer prevention. Several experiments in mouse models have also been investigated to analyze the antitumor activity of TPS. Pharmacological experiments in mice with gastric cancer showed that TPS could increase the serum levels of interleukin-2 (IL-2), interleukin-4 (IL-4), interleukin-10 (IL-10), Immunoglobulin M (IgM), Immunoglobulin M (IgG), and Immunoglobulin A (IgA) in mice and reduce the level of MDA in gastric tissue and the levels of interleukin-6 (IL-6) and TNF-α in serum, which suggests that TPS might be a potential modulator in the intervention of gastric cancer [132]. Another study by Wang et al., showed that Se-ZYTP could significantly reduce tumor volume and weight in U-2 OS-xenograft model mice [104]. Scoparo et al., found that two TPS interventions from green and black tea significantly reduced mortality in septic mice (40% and 25%, respectively), as well as reduced neutrophil entry into the lungs and tissue damage, which may have been due to the difference in uronic acid content in TPSs [116]. In addition to the solid anticancer activity of TPS in experimental animals, studies have shown that other tea-active substances can have synergistic effects when combined with TPS. Combining polysaccharides with other substances could bring several advantages, such as enhanced lethality, reduced toxicity, increased solubility, and reduced drug resistance and immunogenicity [133]. Wang et al., found that the co-administration of oolong tea TPS and polyphenols had a synergistic effect on inhibiting the proliferation and growth of liver tumors of mice, and the antioxidant and immune levels of mice were shown to be significantly increased [75].

### 5.3. Immunostimulatory Activity

The immune system plays a vital role in defending against pathogen invasion and maintaining human health. Generally speaking, polysaccharides mainly regulate the body’s immunity through two pathways: one is to kill pathogens directly, and the other is to enhance the immune system by improving the activity of macrophages and T lymphocytes [134]. Immunostimulatory activity is one of the most remarkable biological functions of natural polysaccharides, and it is related to their active role as a critical basis for antitumor effects [39,135]. Numerous studies have demonstrated the potential regulatory impacts of TPS on immune system stimulation and activation. Monobe et al., investigated the phagocytic activity of macrophages after crude TPS treatment [118]. They found that crude TPS enhanced macrophage activity by stimulating Toll-like receptor 7 (TLR7) and that immature (not fermented by microorganisms) TPS had higher immunostimulatory activity than mature TPS. In addition, studies have found that high-Mw TPS had a stronger immunostimulatory activity than low-Mw TPS. Hu et al., explored the effect of TPS on the immunostimulatory activity of broilers, and they discovered that TPS could significantly increase the serum IgG level; thymus index; serum CAT, GPx, and SOD activities; macrophage activity; and lymphocyte transformation rate in broilers [118]. Yuan et al., studied the synergistic effects of selenium-enriched green tea polysaccharide (Se-TPS) and Huo-ji polysaccharide (HJP) on immune stimulation [9]. The results showed that they could enhance the body’s free radical scavenging ability, improve immune function, and reduce oxidative stress, thereby exhibiting solid immunostimulatory activity. Sun et al., found that two Fuzhuan tea TPSs obtained with hot water and alkali-assisted extraction could promote the proliferation and phagocytosis of macrophages and enhance the activity of their signaling enzymes (acid phosphatase) [31]. In addition, they both showed the positive regulation of cyclophosphamide (CTX)-induced immunosuppression in mice by promoting TNF-α, IL-1β, NO release, inhibiting thymus/spleen index reduction, and inhibiting colon fragmentation. Similar results were reported by Yuan et al. [9]. In addition, chemical modification was found to affect the immune activation of TPS. Glycosidase-modified green tea polysaccharide (ETPS) has been reported to significantly increase NK cell activity and serum hemolysin (HML) levels, delay ear swelling, and increase peritoneal macrophage index, peritoneal macrophage phagocytosis, and splenic phagocytosis [119].

### 5.4. Gut Microbiota-Modulating Activity

The structure and homeostasis of the gut microbiome (GM) are closely related to human health, so the GM is known as the “second genome” of the human body [136]. Recently, it has been increasingly recognized that GM composition and homeostasis are disease modifiers, fundamental components of immunity, and functional entities for metabolism, and an imbalanced GM may contribute to various intestinal diseases [137]. The distribution of the GM in the human body can be modulated by diet, which can influence the composition of the GM by altering the gut environment (e.g., pH) or the activity of certain microbial enzymes [138,139,140]. After GM fermentation, dietary products can regulate certain life activities through the metabolic cycle [141]. Generally, TPSs cannot be degraded or absorbed in the upper gastrointestinal tract, as they mainly metabolized and utilized by the GM in the ileum or colon. Not only does TPS affect the structure and diversity of the GM, but its metabolites in the gut, such as SCFAs (especially acetate, propionate, and butyrate), can also have beneficial effects on gut health [142]. Yang et al., used 16S rDNA amplicon sequencing and metabolomics to analyze the protective effect of Fuzhuan brick tea polysaccharide (FBTP) on dextran sulfate sodium (DSS)-induced ulcerative colitis (UC) in mice [143]. The results showed that the oral administration of FBTP reduced the disease activity index (DAI), prevented colon shortening, and alleviated colon tissue damage and inflammation in UC mice. Furthermore, FBTP intervention also promoted the proliferation of beneficial microbiota (such as *Lactobacillus* and *Akkermansia*) and significant increases in SCFAs, especially butyrate content in the cecum. Butyrate, a type of SCFA, has anti-inflammatory properties and can be absorbed by colon cells as an energy source [144]. This report suggests that FBTP may exert its anti-inflammatory effects by producing SCFAs while improving UC by promoting beneficial bacterial abundance to repair the intestinal epithelial barrier and reduce immune stress [120]. In addition, FBTP intervention was shown to improve tryptophan metabolism in the gut [143]. Tryptophan can be decomposed into indole derivatives by the gut microbiota, which further activates the immune system and ultimately affects the integrity of the intestinal barrier in mice [145]. Wu et al., found that acid TPS from Wuyi rock tea could significantly alter the composition of intestinal flora and improve the dysbiosis of microbial structure in type 2 diabetic rats [121]. Sun et al., reported that the early intake of FBTPs was beneficial to the secretion and mRNA expression of mucin 2, occludin, and ZO-1, thereby preventing and relieving CTX-induced intestinal mucosal damage and protecting intestinal barrier function. In addition, FBTP could improve gut microbiota composition and then promote the proliferation of beneficial bacteria (especially *Lactobacillus*) and the production of SCFAs [31]. Bai et al., established a mouse model of immunosuppression induced by cyclophosphamide and explored the regulatory effect of FBTPS on its immune function and gut microbiota [122]. They found that crude and pure FBTPS could improve the immune organ index, immune cytokines, and immunoglobulin A levels in mice to play an immunoregulatory role. In addition, intestinal injury was ameliorated by improving intestinal morphology and ZO-1 expression. Additionally, they modulated the gut microbiota structure by increasing the relative abundance of Muribaculaceae and decreasing the abundance of *Lachnospiraceae*, *Helicobacteraceae*, and *Clostridaceae*. Chen et al., also used FBTPS as a raw material to study the effect of FBTPS on metabolic syndrome (MS) and gut microbiota dysbiosis in high-fat diet (HFD)-fed mice. The results showed that FBTPS treatment could increase the phylogenetic diversity of the HFD-induced microbiota and significantly inhibit the HFD-induced increase in the relative abundance of pathogenic bacteria such as *Erysipelotrichaceae*, *Coriobacteriaceae*, and *Streptococcaceae*. In addition, FBTPS may also play a key role in the prevention of MS by significantly affecting 44 key OUTs that are negatively or positively correlated with MS [8]. A study by Chen et al., showed that the long-term intake of Longjing tea flower polysaccharide (TFPS) is beneficial to the protection of the intestinal barrier and could promote increases in the number of beneficial microorganisms and their metabolites, thereby maintaining intestinal health [123].

### 5.5. Glucose and Lipid Metabolism-Regulating Activity

Diabetes mellitus (DM) and obesity caused by long-term glucose and lipid metabolism disorders are the two most common and complex metabolic diseases, and their morbidity, chronic course, and disabling complications are increasing [3]. Controlling postprandial blood glucose and inhibiting oxidative stress are considered effective methods for treating diabetes [4]. One treatment for reducing postprandial hyperglycemia is to delay glucose absorption by inhibiting carbohydrate hydrolase enzymes in the digestive organs, such as alpha-amylase and alpha-glucosidase [146]. In China and Japan, coarse tea was used to treat diabetes, and its hypoglycemic activity increased with increasing TPS content in coarse tea [147]. Studies have shown that the hypoglycemic effect of polysaccharides can mainly be implemented through oral or injection therapy. When polysaccharides are administered via injection, they can directly act on target cells through blood circulation; when polysaccharides are orally administered, they usually enter the intestine directly to be decomposed and absorbed due to the high Mw of polysaccharides, thereby exerting indirect hypoglycemic effects [76]. Many studies has revealed the hypoglycemic activity of TPS. Xu et al., extracted TPSs (PTPS-1, PTPS-3, and PTPS-5) from Pu-erh tea with different fermentation years and then compared their effects on postprandial blood glucose in alloxan-induced diabetic mice [36]. The results showed that all PTPSs significantly inhibited postprandial blood glucose in diabetic mice (*p* < 0.05). PTPS-5, with the most prolonged fermentation period, had the strongest inhibitory effect on postprandial hyperglycemia in diabetic mice, and it had no significant difference with acarbose (*p* > 0.05). Similar results were also reported by Deng et al., [125]. In addition, Wei et al., found that TFPS could effectively reduce alloxan-induced hyperglycemia in Sprague-Dawley (SD) rats, maybe due to the superior hydrogen-donating capability of TFPS, which can protect cell membranes from peroxidative damage and alleviate oxidative stress [124]. Altogether, the possible mechanisms of the antidiabetic activity of TPS include: (1) preventing the hydrolysis and absorption of carbohydrates by inhibiting the activity of digestive enzymes in the digestive tract; (2) regulating the structure and diversity of gut microbiota, thereby affecting the types and levels of its metabolites and then regulating intestinal homeostasis and body inflammation; (3) affecting the interaction of the “gut–liver axis”, thereby promoting glucose and lipid metabolism and improving insulin resistance; (4) improving β-cell dysfunction and promoting insulin secretion; and (5) improving oxidative stress and oxidative damage [148,149].

The weight loss and lipid-lowering activities of TPS have also been widely reported. Xu et al., investigated the effects of TPS, caffeine, polyphenols, and the TPS–polyphenol complex in green tea on high-fat diet-fed rats. The results showed that TPS and polyphenols presented apparent blocking effects on fat accumulation and weight gain, and they could inhibit the absorption of fatty acids, reduce serum leptin and blood lipid levels, and reduce the protein expressions of IL-6 and TNF-α. Additionally, a synergistic effect of TPS and polyphenols in lipid-lowering activity was also observed [126]. The weight loss mechanisms of black tea TPS and polyphenols were investigated in male, obese, Sprague-Dawley rats. The results showed that black tea TPS and polyphenols could increase fecal fatty acid content and reduce body weight, adipocyte size, visceral fat weight, and Lee’s index, thereby achieving weight loss. In addition, the black tea TPS also promoted the expression of multiple genes involved in fat metabolism (such as *Aqp1*, *Ugt2b*, and *Gck*), exerting an obesity-suppressing effect [127]. Wu et al., found that oolong tea TPS could effectively reduce serum leptin and lipid levels in obese rats. In addition, it could also inhibit obesity through the effects of pathways affecting fatty acid biosynthesis, steroid hormone biosynthesis, unsaturated fatty acid biosynthesis, glycerolipid metabolism, and glycerophospholipid metabolism [128]. Mao et al., studied the hypolipidemic effect of Chinese Liupao dark tea polysaccharides (CLTPS). It was found that the body weight gain of the high-fat diet-induced rats was significantly inhibited after four weeks of CLTPS intervention [150]. Additionally, the rats’ serum lipid levels, lipid oxidation, and antioxidant enzyme activities were greatly improved in a dose-dependent manner. Accordingly, CLTPS was shown to be able promote the bile acid synthesis pathway and cholesterol catabolism to further prevent atherosclerosis.

### 5.6. Others

In addition to the above-mentioned activities, there have also been some reports on the excellent regulation of TPS in anticoagulant, antibacterial, antifatigue, and skincare activities. Cardiovascular diseases, including cerebral/pulmonary thrombosis and stroke, have recently become the leading causes of death in patients [151]. Of the mentioned activities, anticoagulants can effectively prevent thrombus formation by inhibiting physiological coagulation [134]. Cai et al., investigated the anticoagulant effect of purified fractions of green tea TPS (TPS-1, TPS-2, TPS-3, and TPS-4). It was found that the heterogeneous acidic polysaccharide TPS-4 could exert anticoagulant activity by significantly inhibiting the endogenous and common coagulation pathways of fibrinogen-to-fibrin conversion [16]. Regarding the bacteriostatic activity of polysaccharides, previous reports have shown that polysaccharides have anti-biofilm properties and that they might also inhibit bacterial growth by blocking the input of nutrients [152]. Cai et al., found that green tea polysaccharide conjugate (gTPC) had an antibacterial effect on *Escherichia coli* (*E. coli*) [130]. The morphology of *E. coli* cells treated with gTPC was found to be significantly changed, and some of the *E. coli* cell walls had cytoplasmic leakage due to rupture. At the same time, the permeability of the cell membrane was increased, and the level of intracellular ROS was also significantly increased. A further study revealed that the destruction of the cell wall may be the critical mechanism for gTPC to exert its antibacterial effect. The anti-fatigue activity of Ziyang selenium-enriched green tea (Se-TP) was investigated by Chi et al., [22], who found that Se-TP treatment could prolong the fatigue time of fatigue model mice; reduce serum urea, blood lactate, and lactic dehydrogenase levels; and increase the antioxidant capacity. The anti-fatigue effect of Se-TP may be achieved through enhancing GPx activity to effectively inhibit lipid peroxidation. The appearance of the skin can often provide a rough estimate of a person’s age and health [153]. Water retention is an essential function of skincare cosmetics, and the skin’s moisturizing tissue is damaged with age and exposure to the external environment. Furthermore, ultraviolet (UV)-induced photoaging is the primary factor of the numerous external factors that promote skin aging. Wei et al., studied the protective effects of TPS and TPP on the skin from four perspectives: water absorption and retention, sun protection, the promotion of fibroblast proliferation, and tyrosinase inhibition [153]. The results showed that both TPS and polyphenols had positive skin protective capabilities; TPS (with higher purity) had better hygroscopicity and moisturizing properties, as well as the capacity to promote the proliferation of fibroblasts, whereas TPP could strongly absorb UV-A and UV-B, thereby better reducing ultraviolet damage to the skin. Accordingly, it also showed potent tyrosinase inhibition. However, further human clinical validation is still required.

## 6. Conclusions and Prospects

As an essential ingredient in tea, TPS has attracted more and more attention due to its excellent bioactivity and great development potential. The authors of this review fully discussed the extraction process, preliminary physicochemical properties, and bioactivities of TPS. Among the preparation methods of TPS, hot water extraction is the most widely reported, but the yield of polysaccharides obtained with this method is low. Therefore, the development of other auxiliary methods combined with hot water extraction can significantly improve the yield of TPS. For the preparation of TPS, the extraction and isolation of active TPS with high purity, maximum extraction yield, and complete structure is a major future focus because the functional food and pharmaceutical industries require simpler, more efficient, and cheaper methods for the large-scale production of high quality TPS. The analysis of physicochemical properties showed that the physicochemical properties of TPS, especially the uronic acid content, molecular weight, and monosaccharide composition, had significant effects on its bioactivity. Thus, more research on the physicochemical properties of TPS (especially regarding the availability of its bioactivities) is needed to broaden its biological applications. Over the past decade, the potential application of TPS in functional foods or medicines has attracted increasing attention due to its excellent bioactivities such as biodegradability, nontoxicity, and biocompatibility. TPS has shown its inherent advantages in intervening in the formation and development of metabolic diseases, anti-tumor activities, and skincare activities. The current study shows that TPS has many excellent bioactivities, but its bioactivity is likely to be significantly affected by the origin of its tea, the variety of its tea trees, its tea-making process, and its extraction method. Therefore, the specific effects of TPS preparation and source on its bioactivity need comprehensive research. In addition, the underlying mechanism of how TPS exerts its favorable bioactivity is unclear. Many reports have revealed that TPS likely acts as a prebiotic, exerting its health care activities through direct interactions with the gut microbiota and indirect effects by affecting the “gut–liver axis” or “gut–pancreas axis”. However, this area still needs further exploration, as all current work is based on cell models or rodent models. Whether a similar function will be observed after human intervention remains unknown. Therefore, clinical trials should be carried out to further evaluate the bioactivity and mechanism of TPS on the basis of animal experiments. It is believed that either the exploration mechanism of its population cohort or the specific exploration of its application scope and conditions will become a hot spot in future work on TPS.

## Figures and Tables

**Table 1:** Comparison of extraction methods of tea polysaccharide (TPS).

Extraction Method	TPS Origin	Extraction Step	Ref
Hot water extraction	Green tea leaves and flowers	Pre-extraction with 95% ethanol at 40 °C for 2 h, repeated three times; a water bath extraction at 60 °C for 2 h, repeated 3 times	[14]
	Fuan Baicha and Pingyang Tezaocha	Extraction at 80 °C for 1.5 h, repeated two times	[15]
	Fuzhuan tea	2 h extraction time, 1:20 solid–liquid ratio, and 95 °C extraction temperature; repeated three times	[10]
	White tea	8 min extraction time, 54.1 °C extraction temperature, 12.48 L/g material–water ratio; repeated four times	[16]
	Green tea	Heating in a water bath at 90 °C for 2 h with continuous stirring	[17]
	Green tea	Pre-extraction with absolute ethanol for 24 h and extraction with deionized water at 60 °C for 90 min	[18]
	Chin brick tea	80% ethanol pretreatment and continuous stirring with distilled water (1:20, *w*/*v*) at 90 °C for 2 h	[19]
	Liupao tea	80% ethanol pretreatment for 24 h and extraction with deionized water at 70 °C for 2 h; repeated three times	[20]
	Tea flowers	Extraction at 90 °C for 1 h (2 times)	[21]
	Green tea	80% ethanol pretreatment at 70 °C for 1.5 h, extraction with ethanol at 40 °C for 3 h	[22]
	Green tea	Pretreatment with two times volume of 95% ethanol at 50 °C for 4 h, 1:8 solid–liquid ratio, and extraction with stirring at 50 °C for 120 min	[23]
	Green tea	Pretreatment with 95% alcohol (1:5, *w*/*v*) for 2 h, extraction in hot water (1:10, *w*/*v*) at 80 °C; repeated 3 times for 1 h each time	[24]
	Green tea	95% ethanol (1:6, *w*/*v*) pretreatment at 60 °C for 4 h and extraction with distilled water (1:10, *w*/*v*) at 80 °C for 4 h; repeated 3 times	[25]
	Keemun black tea	Pretreatment with 95% ethanol (1:6, *w*/*v*) at 80 °C for 2 h and immersed in distilled water (1:10, *w*/*v*) at 80 °C for 4 h; repeated four times	[26]
Ultrasonic-assisted extraction	Low-grade green tea	80 °C extraction temperature, 60 min extraction time, 400 W ultrasonic power, and 22 mL:g liquid–solid ratio	[27]
	Coarse tea	Pretreatment in an ultrasonic bath (50 °C, 200 W) for 30 min followed by extraction in a water bath for 90 min; repeated three times	[23]
	Green tea flowers	Ultrasonic power (25 °C, 100, 150, 200, 250, and 300 W) extraction for 5 min; repeated 2 times	[21]
	Yellow tea	95% ethanol pretreatment for 6 h, 90 °C water bath extraction for 55 min (repeated twice), and sonication (20 kHz, 500 W) for 55 min	[21]
Microwave-assisted extraction	Green, black, and oolong teas	1:20 solid/liquid ratio, 200–230 °C extraction temperature, and 2 min extraction time	[28]
	Green tea flowers	Extraction at controlled microwave power for 5 min followed by extraction with distilled water for 5 min at the same microwave power	[21]
	Green tea	Extraction in a 600 W microwave apparatus for 30 min, followed by stirring in a water bath for 90 min; repeated three times	[29]
Enzymolysis extraction	Green tea	Extraction at 100 °C for 3 h and aqueous extraction with pectinase and tannase at 35 °C for 2 h	[30]
	Green tea	Extraction with complex enzymes (cellulase:pectinase:glucanase = 1:1:2) at 50 °C for 30 min, boiling at 90 °C for 10 min, and then extraction in a water bath at 50 °C for 80 min	[29]
	Green tea leaves and flowers	95% ethanol pretreatment at 40 °C for 2 h (repeated 3 times), treatment with 0.5% (m/v) pentosan complex enzyme solution (45 °C, pH 5.5) for 2 h, and extraction in 45 °C water bath for 2 h	[14]
	Green tea	Heating in a water bath at 90 °C for 2–4 h, repeated twice; incubating with 0.5% pectinase (260,001 PGU/mL, *v*/*w*) at 40 °C for 30 min; and heating at 90 °C for 1 h to inactivate the enzyme	[31]
Hydro/solvothermal extraction	Chinese tea Zhongcha 108	Extraction at 120 °C for 1 h	[1]
Alkali-assisted extraction	Fuzhuan brick tea	Extraction with 0.1 M NaOH solution (pH = 10.0) at 60 °C, repeated 3 times	[32]
Supercritical fluid extraction	Green tea	380 μm particle size, 20% absolute ethanol, 35 MPa extraction pressure, 45 °C extraction temperature, and 2 h extraction time	[33]
Anionic reverse micelle extraction	Green tea	pH = 4.6, 0.06 M guanidine hydrochloride, 7% methanol, and 0.05 M NaCl; forward extraction	[34]

**Table 2:** Monosaccharide composition of different TPSs.

TPS Origin	Monosaccharide Composition and Molar Ratio	Ref
Green tea	WE, Rha: Ara: Gal: Glc: Xyl: Man: Fru: GalA = 4.11: 9.96: 28.05: 29.22: 3.46: 4.62: 4.14: 16.43, respectively; UAE, 2.27: 9.22: 27.54: 36.05: 5.38: 4.75: 6.72: 8.07, respectively; MAE, 4.03: 11.84: 27.06: 31.09: 3.64: 6.17: 6.84: 9.33, respectively; EE, 5.40: 8.86: 12.32: 44.24: 3.15: 4.38: 11.78: 9.87, respectively	[21]
Green tea	Ara: Xyl: Fuc: Glc: Gal = 6.49: 2.60: 6.53: 43.27: 41.11, respectively;	[46]
Natural and artificial selenium-enriched green teas	ASe-TPS2, Rha: Ara: Glc: Xyl: GalA = 1.93: 7.05: 1.00: 1.05: 26.12, respectively; NSe-TPS2, Ara: Gal: GluA: GalA = 0.59: 1.00: 0.49: 1.24, respectively	[47]
Selenium-enriched green tea	Se-TPS1, Fuc: Rha: Ara: Gal: Glc: GlcA: GalA = 0.07: 0.21: 0.58: 1.00: 0.47: 0.17: 1.75, respectively; Se-TPS2, Fuc: Rha: Ara: Gal: Glc: GlcA: GalA = 0.07: 0.28: 0.59: 1.00: 0.10: 0.49: 1.24, respectively; Se-TPS3, Fuc: Rha: Ara: Gal: Glc: GlcA: GalA = 0.07: 0.38: 0.72: 1.00: 0.30: 0.19: 0.88, respectively	[48]
Fuzhuan brick tea	FBTPS, Rib: Man: Ara: Rha: Gal: Glc: GlcA: GalA = 1.69: 3.66: 11.83: 12.11: 19.15: 21.97: 1.41: 28.17, respectively	[52]
Fuzhuan brick tea	FBTPS-3, Man: Rha: GalA: Gal: Ara = 8.7: 15.5: 42.2: 19.7: 13.9, respectively	[53]
Green tea	GTP consisting only of Glc	[54]
Yingshan Cloud Mist green tea	GTPS, Rha: Ara: Xyl: Man: Glc: and Gal = 11.4: 26.1: 1.9: 3.0: 30.7: 26.8, respectively	[55]
Selenium-enriched green tea	SeTPS-1, Glc: Gal = 80.1: 2.3; SeTPS-2, Glc: Gal = 80.1: 2.3, respectively	[56]
Yellow tea	YTPS-N, Man: Rib: Rha: GlcA: GalA: Glc: Gal: Ara = 1.65: 1: 10.95: 1.06: 2.03: 5.49: 3.50: 4.02; YTPS-U, 1.72: 1: 11.05: 1.09: 2.13: 5.36: 3.62: 4.17, respectively	[26]
Large-leaf yellow tea	LYTP, Ara: Gal: GalA: Rha: Glc: GlcA: Man	[57]

**Table 3:** In vivo bioactivity of different TPSs.

Bioactivity	TPS Origin	Regulatory Mechanism	Ref
Antioxidant and hepatoprotective activity	Ziyang green tea	Ameliorating high-fructose diet-induced pancreatic β-cell damage and inhibiting hepatic steatosis and oxidative damage	[112]
	Ziyang green tea	Mediating antioxidant and free radical scavenging, thereby effectively preventing liver damage	[113]
	Green tea	Promoting superoxide dismutase (SOD), catalase (CAT), and glutathione peroxidase (GPx) activity in blood, liver, and heart	[114]
	Huangshan Maofeng	Inhibiting lipid peroxidation while promoting the body’s antioxidant activity to protect the liver	[23]
	Longjing 43 tea flower	Inhibiting the elevation of serum aspartate aminotransferase (AST) and alanine aminotransferase (ALT) levels, reducing the formation of malondialdehyde (MDA), and simultaneously enhancing the activities of SOD and GPx to reduce liver damage	[98]
	Keemun black tea	Improving the enzymatic and non-enzymatic antioxidant defense system to protect the liver, thereby effectively alleviating the production of free radicals in the body and inhibiting lipid peroxidation in liver tissue	[24]
Antitumor activity	Dark brick tea	Inhibiting cancer cell proliferation and migration and inducing cancer cell apoptosis	[103]
	Ziyang green tea	Inhibiting the proliferation of human osteosarcoma cells (U-2 OS)	[104]
	Green tea	Targeting lysosomes and activated caspase-9/-3 via the lysosome-mitochondrial pathway to induce apoptosis in colon cancer cells (CT26)	[105]
	Tea flowers	Inhibiting the proliferative activity of human gastric cancer cells (BGC-823)	[98]
	Green tea	Increasing the levels of SOD, CAT, and GPx while inhibiting lipid peroxidation and pro-inflammatory cytokine levels from attenuating oxidative damage and inflammatory responses	[115]
	Ziyang green tea	Inhibiting the proliferation of osteosarcoma cells in vitro and the growth of tumor volume and tumor weight in vivo	[104]
	Green and black teas	Inhibiting pulmonary neutrophil recruitment and oxidative tissue damage, resulting in higher anti-inflammatory effects and resistance to murine sepsis	[116]
	Oolong tea	Inhibiting tumor growth, reducing liver toxicity and nephrotoxicity, stimulating the body’s antioxidant activity and immune function, and finally achieving an anti-liver cancer effect	[75]
	Green tea	Increasing the Bcl2-associated X protein (Bax)/B-cell lymphoma-2 (Bcl-2) ratio, elevating caspase-3 protein expression, and decreasing miR-93 expression in prostate cancer cells	[54]
Immunostimulatory activity	Green tea	Activating the TLR7 receptor and enhancing the macrophage activity	[117]
	Green tea	Improving the serum IgG level, thymus index, macrophage activity, and lymphocyte transformation rate in broilers, as well as increasing the serum antioxidant enzyme activity	[118]
	Selenium-enriched green tea	Enhancing the regulatory mechanism involved in free radical scavenging, synergistically improving immune function, and reducing oxidative stress	[9]
	Green tea	Enhancing the body’s cellular immunity and humoral immunity	[119]
	Fuzhuan brick tea	In vitro: promoting the in vitro proliferation activity and phagocytic capability of macrophages and enhancing the activity of acid phosphatase; in vivo: promoting the release of tumor necrosis factor (TNF-α), interleukin-1β (IL-1β), and nitric oxide (NO) and then inhibiting decreases in thymus/spleen index and colon rupture	[30]
	Selenium-enriched green tea	Improving the spleen and thymus index, promoting the lymphocyte proliferation and NK cell activity in the spleen, promoting the CD4 T cell proliferation, and reducing oxidative stress	[9]
Gut microbiota modulating activity	Fuzhuan brick tea	Reducing the disease activity index (DAI) in mice with enteritis, alleviating the colonic tissue damage and inflammation, and simultaneously promoting the proliferation of beneficial gut microbiota and the increase in short-chain fatty acids (SCFAs)	[120]
	Wuyi rock tea	Improving gut microbiota composition and microbial structural dysbiosis in type 2 diabetic rats	[121]
	Fuzhuan brick tea	Promoting the secretion and mRNA expression of mucin 2, occludin, and zonula occludens 1 (ZO-1); altering gut microbiota composition; and stimulating the proliferation of beneficial bacteria and production of SCFAs	[30]
	Fuzhuan brick tea	Increasing the phylogenetic diversity of the gut microbiota, suppressing the increase in the relative abundance of pathogenic bacteria, and altering key OUTs associated with metabolic syndrome	[8]
	Fuzhuan brick tea	Altering the gut morphology and ZO-1 expression, increasing the relative abundance of Muribaculaceae, and decreasing the relative abundance of *Lachnospiraceae*, *Helicobacteraceae*, and *Clostridaceae*	[122]
	Tea flowers	Protecting the intestinal barrier function and promoting the increase in the number of beneficial microorganisms and their metabolites, thereby maintaining intestinal health and improving adaptive intestinal immunity	[123]
Glucose and lipid metabolism-regulating activity	Pu-erh tea	Inhibiting intestinal alpha-glucosidase activity	[36]
	Green, oolong, and black teas	Enhancing in vitro free radical scavenging activity and α-glucosidase inhibition in skeletal muscle cells	[76]
	Tea flowers	Protecting cell membranes from peroxidative damage and reducing oxidative stress	[124]
	Pu-erh tea	Inhibiting the intestinal alpha-glucosidase activity	[125]
	Green tea	Adjusting body weight, reducing serum triglyceride (TG) and leptin (LT) levels, inhibiting fatty acid absorption, improving anti-inflammatory activity, and treating obesity	[126]
	Black tea	Inhibiting the formation and accumulation of fat, promoting the decomposition of fat, and promoting the expression of essential genes involved in fat metabolism	[127]
	Oolong tea	Decreasing serum LT levels in obese rats, improving blood lipids and antioxidant levels, and affecting lipid metabolism pathways	[128]
	Fenggang zinc selenium tea	Improving oxidative stress, inhibiting lipid peroxidation, and enhancing liver protection	[129]
Anticoagulant activity	Green tea	Inhibiting the intrinsic and common coagulation pathways of fibrinogen-to-fibrin conversion without inhibiting the extrinsic pathway	[16]
Bacteriostatic activity	Green tea	Destroying the cell wall of *Escherichia coli* and increasing the permeability of the cell membrane and the content of intracellular ROS	[130]
Anti-fatigue activity	Ziyang green tea	Preventing lipid peroxidation by modification of GPx activity	[22]
Skincare activity	Green tea	Promoting skin’s moisturization and enhancing fibroblast proliferation capability	[11]

## Data Availability

Not applicable.
